# Benefits of maternally-administered infant massage for mothers of hospitalized preterm infants: a scoping review

**DOI:** 10.1186/s40748-023-00151-7

**Published:** 2023-05-03

**Authors:** Dana B. McCarty, Sandra Willett, Mary Kimmel, Stacey C. Dusing

**Affiliations:** 1 Department of Health Sciences, University of North Carolina at Chapel Hill, 3024 Bondurant Hall, CB#7135, Chapel Hill, NC 27599-7135 USA; 2 Department of Physical Therapy, Munroe-Meyer Institute, University of Nebraska Medical Center, Omaha, USA; 3 Department of Psychiatry, University of North Carolina at Chapel Hill, Chapel Hill, USA; 4 Division of Biokinesiology and Physical Therapy, University of Southern California, Los Angeles, USA

**Keywords:** Infant massage, Mother, Parent, Neonatal Intensive Care, Maternal mental health, Anxiety, Depression, Maternal-infant interaction

## Abstract

**Objectives:**

Infant massage (IM) is a well-studied, safe intervention known to benefit infants born preterm. Less is known about the benefits of maternally-administrated infant massage for mothers of preterm infants who often experience increased rates of anxiety and depression in their infants’ first year of life. This scoping review summarizes the extent, nature, and type of evidence linking IM and parent-centered outcomes.

**Methods:**

The Preferred Reporting Items for Systematic reviews and Meta-Analyses Extension for scoping reviews (PRISMA-ScR) protocol was followed using three databases: PubMed, Embase, and CINAHL. Thirteen manuscripts evaluating 11 separate study cohorts met pre-specified inclusion criteria.

**Results:**

Six primary topics related to the influence of infant massage on parent outcomes emerged: 1) anxiety, 2) perceived stress, 3) depressive symptoms, 4) maternal-infant interaction, 5) maternal satisfaction, and 6) maternal competence. Emerging evidence supports that infant massage, when administered by mothers, benefits mothers of preterm infants by reducing anxiety, stress, and depressive symptoms and improving maternal-infant interactions in the short-term, but there is limited evidence to support its effectiveness on these outcomes in longer periods of follow-up. Based on effect size calculations in small study cohorts, maternally-administered IM may have a moderate to large effect size on maternal perceived stress and depressive symptoms.

**Conclusions:**

Maternally-administered IM may benefit mothers of preterm infants by reducing anxiety, stress, depressive symptoms, and by improving maternal-infant interactions in the short-term. Additional research with larger cohorts and robust design is needed to understand the potential relationship between IM and parental outcomes.

## Introduction

The risk of preterm birth in the U.S. exceeds 10%, the highest rate among developed nations [1]. These infants require prolonged medical care in the Neonatal Intensive Care Unit (NICU), introducing grief, uncertainty, and stress into the parent’s new caregiver role. Over 60% of mothers of preterm infants demonstrate depressive symptoms, and over 70% demonstrate symptoms of anxiety during their infant’s hospitalization [2]. Such maternal symptoms are associated with short and long-term negative outcomes related to parent-infant bonding behaviors, infant temperament, breastfeeding, infant health and motor outcomes, and adolescent conduct behavior [3].

Multiple interventions are designed to address anxiety, depression, and well-being for parents of preterm infants [4]. Interventions like parent education, psychotherapy, and cognitive behavioral therapy demonstrate promise in improving symptoms, but supporting studies often lack robust research methodology to make definitive conclusions about effectiveness [5]. NICUs also face challenges to implementing such programs for parents, most of which rely on multidisciplinary contributions, in sustainable ways [6]. Given the high rate of preterm birth in the US, finding cost-effective solutions to support maternal mental health during the hospitalization and follow up period is of utmost importance.

Whether administered by medical professionals or parents, infant massage (IM) in the NICU is a safe intervention with established infant benefits including improved weight gain, improved sleep quality, reduced muscle tone, and improved oral feeding [7]. Early research in IM focused solely on delivery by a professional that was provided at high frequencies, often multiple times a day or week [8]. More recent research of IM in the hospital setting has incorporated the parent as the primary administrator of IM, but still with a greater focus on infant outcomes than parent outcomes [7]. NICUs have been slow to incorporate IM into regular standard of care for hospitalized infants. Reasons for lack of uptake include healthcare practitioner concerns about maintaining workload capacity and interference with daily cares – especially given the high frequency of massage delivery in protocols that have been studied [7, 8]. Additional concerns around wide-scale IM implementation relate to the importance of maintaining cluster care, discerning infant medical fragility, and individualizing massage based on infant cues and response [9]. However, if parents are trained by professionals to implement IM with sensitivity to infant cues and readiness [10, 11], there is potential that this intervention could be provided by the parent with oversight from the medical team.

Maternally-administered IM has also demonstrated benefits to mothers of non-hospitalized, *fullterm* infants in reducing depressive symptoms [12], increasing maternal-infant interaction [13], and promoting more positive parenting attitudes [14]. Considering the wealth of support for IM benefitting fullterm infants, preterm infants, and parents of fullterm infants, it is important to understand the extent to which the relationship between IM and parenting outcomes has been examined in the context of Neonatal Intensive Care – a time of heightened parent mental health challenges.

### Objective

This paper reports findings from a scoping review of studies that collected parent-centered measures related to implementation of parent-administered IM during the NICU. We summarize the extent, nature, and type of evidence linking IM and objectively measured parent-centered outcomes of any kind in order to better understand potential benefits of IM for parents of preterm infants.

## Methods

### Eligibility criteria

The following inclusion criteria were used to select studies: 1) published in a peer-reviewed journal, 2) published in English, 3) IM administered exclusively in hospital settings by a parent (biological, non-biological, mother, or father), 4) studies reported quantitative outcome measures, 5) outcomes related to mother or parent, 6) study categorized as a clinical trial, or used secondary data from a clinical trial. Exclusion criteria included: 1) dissertations, book chapters, and meeting abstracts, 2) studies conducted in the outpatient setting or exclusively with fullterm infants, and 3) studies that only assessed infant-centered outcomes.

### Data sources and search strategy

The protocol for the scoping review was drafted using the Preferred Reporting Items for Systematic Reviews and Meta-analysis Extension for scoping reviews (PRISMA-ScR) [15], The final protocol was registered prospectively with the Open Science Framework [16]. Three databases were systematically searched: PubMed, Embase, and CINAHL. Searches were completed during August of 2022, and therefore all studies up until this month of publication were included. References from included articles were also screened for inclusion. Search terms were grouped under three main categories: intervention-related, parent-related, and setting-related. Because of the author’s (DM) knowledge of two infant programs that include IM as components (Auditory, Tactile, Visual, and Vestibular (ATVV) [17] (now known as Massage + intervention) [18] and Supporting and Enhancing NICU Sensory Experiences (SENSE) [11], these programs were specifically named as part of the search strategy. A search strategy using keywords was developed by the primary author (DM) in consultation with a university librarian and included (("infant massage" OR "ATVV" OR “SENSE”) AND ("Neonatal Intensive Care") AND (“parent” OR “mother”)). Although “parent” was used with the intent of ensuring that studies examining both maternal and paternal outcomes were included in the analysis, only one study enrolled fathers [19]. Secondary searches involved scanning publication reference lists and the “related articles” feature of PubMed for eligible articles, and four additional articles were included using this method. Results were imported to Covidence, a systematic review production tool for title/abstract/full-text review and data abstraction [20].

### Data extraction

Two reviewers (DM and SW) independently reviewed and extracted papers that met inclusion criteria for full text review. Any disagreement between the two reviewers about papers to include for full text review resulted in full text review of the paper in question. Papers that passed full-text review were evaluated with an extraction table designed to collect the following study characteristics: study aims, study design, data sources, study population, intervention characteristics, data analysis strategy, outcome measures, results, implications, strengths, and limitations. Data extracted were then reviewed using a descriptive approach to summarize key findings.

### Quality assessment

Risk of bias was assessed using the RoB:2 revised Cochrane risk-of-bias tool for randomized trials [21]. Full agreement between reviewers (DM and SW) was reached after discussion. See Table 1.

**Table 1:**
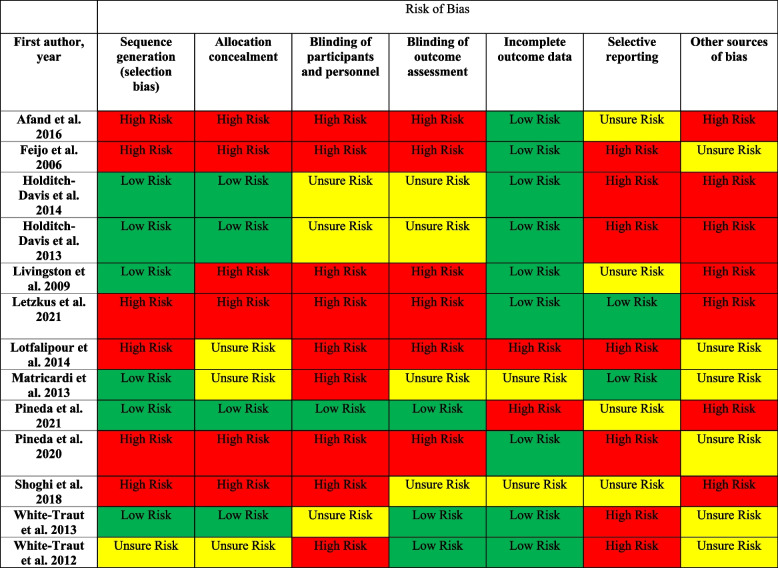
Quality assessment, Afand et al. 2017 [22], Feijo et al. 2006 [23], Holditch-Davis et al. 2014 [24], Holditch-Davis et al. 2013 [25], Livingston et al. 2009 [26], Letzkus et al. 2021 [27], Lotfalipour et al. 2019 [28], Matricardi et al. 2013 [19], Pineda et al. 2021 [11], Pineda et al. 2020 [29], Shoghi et al. 2018 [30], White-Traut et al. 2013 [31], White-Traut et al. 2012 [32]

## Results

### Study selection and characteristics

**Fig. 1:**
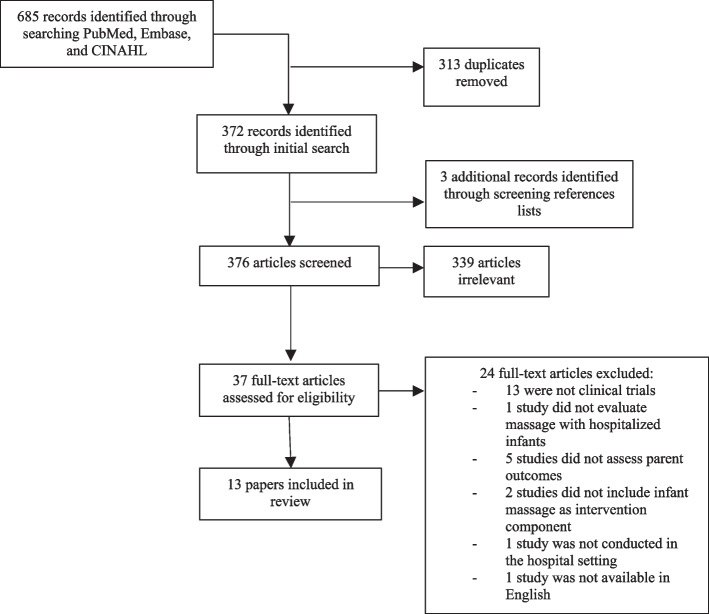
Flow diagram of the research selection process

The initial search identified 685 articles. Three-hundred and thirteen duplicates were removed, and the remaining 372 article titles and abstracts were screened for suitability based on inclusion and exclusion criteria. Three additional articles were found based on article reference review. Upon full-text review, 2 studies were excluded after the description of the intervention did not include explicit mention of IM despite having been referenced by another article as being a program that did include parent-administered IM [33]. In total, full-text review was completed for 37 articles, and 13 met all eligibility criteria (Fig. 1).

Thirteen manuscripts evaluating 11 separate study cohorts were included. Study designs included a feasibility pilot (*n* = 1), prospective intervention group vs. historical control group (*n* = 1), quasi-experimental with intervention group vs. control group (*n* = 1), and RCT (*n* = 10). See Table 2 for further details.

**Table 2:** Study characteristics

First author, year	**Study design**	**N**	**Sample Characteristics**	**Intervention type**	**Intervention description**	**Control group/Alternative Intervention**	**Outcome measures**	**Results**
**Afand et al. 2017** [22]	RCT	75	Primiparous subjects, age 18–40 years, no mental illness, with infants 32–37 weeks gestational at birth	Massage only	Field Method: 8-min massage session including two similar standard 4-min parts; in the first 4-min by the researcher while the mother was observing and for the second four minutes, the mother performed the massages	Yes	STAI	The mean scores of maternal state anxiety (MSA) in mothers of the massage group were significantly lower than control group In before and after comparison, the mean score of MSA in two groups was significantly decreased
**Feijo et al. 2006** [23]	RCT	20	Mothers of infants < 37 weeks, medically stable, to be discharged within 24 h Racial/Ethnic Characteristics: Not reported	Massage only	Field Method: Massage was performed in an 8-min session including two similar standard 4-min parts	Control group observed their infant being massaged	STAI, POMS, Infant Massage Questionnaire	A significant Time by Group interaction effect was noted for STAI indicating that only the mothers who conducted the preterm infant massage reported decreased anxiety. A significant main effect of Time was noted for POMS indicating that both groups experienced a decrease in depressed mood
**Holditch-Davis et al. 2014 **[24]	3-arm RCT	240	Mothers of infants who weighed less than 1750 g at birth without history of bipolar disorder or major depressive disorder Racial/Ethnic Characteristics: Black = 68.3% White = 19.2% Hispanic = 8.3% Other = 4.2%	Massage as component	ATVV intervention: 10 min of auditory, tactile (moderate touch stroking or massage) and visual stimulation, followed by 5 min of vestibular stimulation	Kangaroo Care group and control group	CESD, STAI, PPQerinatal PTSD, PSS:PBC, The Worry Index, The Vulnerable Child Scale, The HOME Inventory	Mothers who massaged had a more rapid decline in depressive symptoms that leveled out earlier than mothers not in engaging in any intervention; Parenting stress was lower for mothers who engaged in any intervention than those who did not. Mothers who performed massage had higher HOME scores than mothers who engaged in neither
**Holditch-Davis et al. 2013 **[25]	3-arm RCT	240	Mothers of infants who weighed less than 1750 g at birth without history of bipolar disorder or major depressive disorder Racial/Ethnic Characteristics: Black = 68.3% White = 19.2% Hispanic = 8.3% Other = 4.2%	Massage as component	ATVV intervention: 10 min of auditory, tactile (moderate touch stroking or massage) and visual stimulation, followed by 5 min of vestibular stimulation	Kangaroo Care group and control group	Satisfaction Survey	No significant differences occurred between the groups in the subscaleor on the three global items: whether the mother would recommend the study to others, the degree to which she changed as a person, and the degree to which she changed as a mother. On the intervention satisfaction subscale at discharge, ATVV mothers had significantly higher scores on Item #8 (learn new ways to stimulate and teach my infant) than did the other two groups
**Livingston et al. 2009 **[26]	RCT	12	Mothers of infants with a minimal gestation of 32 weeks, intact skin and were able to receive massage as determined by the attending neonatologist	Massage only	Total of 20 min, beginning and ending with 5 min of containment, and the middle 10 min consisting of infant massage, which included stroking of the skin (arms, legs, stomach, chest, back, face and head)	Yes	Caregiver satisfaction survey, BDI-II	Both groups demonstrated a decrease in depressive Symptoms All caregivers in the massage group reported high levels of satisfaction Other measures of satisfaction (e.g. caregiver’s comfort while massaging infant, caregiver’s comfort while holding infant) remained equally high at day 7 and at the 1- month follow-up
**Letzkus et al. 2021 **[27]	Single cohort, feasibility pilot	12	English-speaking mothers of infants ≤ 32 weeks gestation at birth without social circumstances precluding maternal presence at bedside	Massage as Component	Intervention bundle was composed of 5 evidence-based practice interventions that included massage. It was recommended that massage be performed 15 min twice a day and at least 2 h apart between sessions	None	PSS-NICU, PROMIS-Anxiety Self-report diary	PSS-NICU revealed a decrease in the total score from 7.4 ± 0.8 prior to intervention start to 5.7 ± 0.7 prior to NICU discharge (*P* = .02) Participation in the intervention bundle did not result in increased anxiety. A decrease in the depression raw scores was noted in participating mothers (11.1 ± 0.9 prior to intervention start vs 9.0 ± 0.5 prior to NICU discharge; *P* = .002, paired t test)
**Lotfalipour et al. 2019** [28]	RCT	52	Mothers of infants 30–37 weeks gestational age at birth	Massage only	15 min total massage in 3 phases lasting 5 min each	Yes	POMS	Mean mood scores of mothers with preterm infants were not significantly different between the control and intervention groups before massage, but was significantly different after the intervention
**Matricardi et al. 2013 **[19]	RCT	42	Mothers and fathers of singleton infants ≤ 32 weeks gestational age	Massage as component	Field Method: Two 10 min sessions of moderate pressure strokes followed by kinesthetic stimulation	Yes	PSS-NICU	Mothers reported more stress compared with fathers, above all for PRA. A parental intervention was effective in reducing stress-role alteration in mothers, but not fathers
**Pineda et al. 2021 **[11]	RCT	70	Parents of infants ≤ 32 weeks gestation Racial/Ethnic Characteristics: Black = 44%	Massage as component	5 modes of sensory intervention: Depending on infant's age and individual tolerance, tactile interventions employed included gentle human touch, skin-to-skin care; weekly modifications made if necessary	Yes	PSS-NICU, STAI, EPDS, PPQ, MCQ, BDI-II	No differences between standard-of-care and treatment groups for parent outcome measures reached significance at term equivalent age or at one year follow up
**Pineda et al. 2020 **[29]	Prospective quasi-experimental with historical controls	30	Mothers of infants ≤ 32 weeks gestation Racial/Ethnic Characteristics: Black = 41%	Massage as component	5 modes of sensory intervention: Depending on infant's age and tolerance, tactile interventions employed included gentle human touch, skin-to-skin care or massage with a targeted minimum of 3 h by term equivalent age	Historical control that received standard of care	PSS, STAI, EPDS, PPQ, Parental Role Alterations subscale from PSS, MCQ	Mothers who received the SENSE program had more confidence, measured by the MCQ after controlling for infant and maternal factors There were no other relationships between group assignment and any of the other maternal factors
**Shoghi et al. 2018 **[30]	RCT	40	Mothers of late preterm infants (34–37 weeks gestational age)	Massage only	A head-to-toe massage was given to fully naked or diapered neonates over 15 min	Yes	Maternal Attachment Behaviors Scale	The study showed a statistically significant difference between baseline and postintervention in maternal attachment behaviors for both groups. A significant between group difference existed postintervention for maternal attachment between intervention and control groups
**White-Traut et al. 2013 **[31]	RCT	198	Mothers of infants 29–34 weeks gestation 2 + risk factors (eg., minority, low education) Racial/Ethnic Characteristics: Black = 50% Hispanic = 50%	Massage as component	(1) twice-daily infant stimulation using the ATVV (auditory, tactile, visual, and vestibular-rocking stimulation) and (2) four maternal participatory guidance sessions plus two telephone calls by a nurse-community advocate team	Parent Education Program	NCAST, DMC	Results identified a trend toward more positive mother-infant interaction during both feeding and play for dyads who received the H-HOPE intervention compared to those in the attention control group, and these differences were significant or marginally significant when covariates were controlled
**White-Traut et al. 2012 **[32]	RCT	26	Mothers of infants < 1750 g at birth without history of significant mental illness Racial/Ethnic Characteristics: Black = 69% White = 15% Other16%	Massage as component	ATVV intervention: 10 min of auditory, tactile (moderate touch stroking or massage) and visual stimulation, followed by 5 min of vestibular stimulation	Kangaroo Care group	Infant engagement and disengagement cues	ATVV elicited more disengagement than did kangaroo care. Separate analysis of the subtle and potent behavioral cues revealed that the ATVV intervention elicited significantly more potent engagement, subtle disengagement, and potent disengagement behaviors from infants

*STAI* State-Trait Anxiety Inventory, *POMS* Profile of Mood States, *CESD* Center for Epidemiologic Studies Depression Scale, *PPQ* Perinatal Post Traumatic Stress Disorder Questionnaire, *PSS:PBC* Parental Stress Scale: Prematurely Born Child, *BDI-II* Beck Depression Inventory II, *PROMIS-anxiety* Patient-Reported Outcomes Measurement Information System, *PSS:NICU* Parental Stress Scale:Neonatal Intensive Care Unit, *PSS* Parental Stress Scale, *MCQ* Maternal Confidence Questionnaire, *EPDS* Edinburgh Postnatal Depression Scale, *ATVV* Auditory, Tactile, Visual, Vestibular Intervention, *NCAST* The Nursing Child Assessment Satellite Training-Feeding Scale, *DMC* Dyadic Mutuality Code

A total of 910 parents, 891 of which were mothers, were enrolled across studies. Studies were based in the US (*n* = 10) [11, 23–25, 29, 31, 32], Iran (*n* = 3) [22, 28, 30], and Italy (*n* = 1) [19]. Participant numbers ranged from 11 to 240 mother-infant dyads. Within the 10 US-based manuscripts [11, 23–25, 29, 31, 32], maternal age ranged from 18 to 39 years and distribution of race, ethnicity was reported as: White, non-Hispanic 10–66%; Black, non-Hispanic 20–72%; Hispanic or other 10–51.5%. Two small US-based studies did not report maternal race, ethnicity, or age [26, 27]. Additional sociodemographic information collected included maternal education (*n* = 11, range of < 8–15 years) [11, 23–25, 29, 31, 32], annual income of < $25,000 (*n* = 2, 45-50%) [29], and economic status (*n* = 1, poor/low = range of 14.2-22%) [22]. Two studies included the Hollingshead four-factor index of socioeconomic status. One of these studies reported the full measure (range of 42–82 on 0–90 scale) [19] while the other reported an un-named subscale of the measure (group means of 3.3 and 3.4) [23].

Shared criteria for infant eligibility specified that all infants were 1) hospitalized, 2) medically cleared to participate in massage (although criteria varied across studies), and 3) < 37 weeks gestational age at birth. Infant gestational ages at birth varied significantly from 21–36 weeks. The earliest gestational or postmenstrual age for infant massage initiation was at 32–34 weeks in medically stable (i.e., not mechanically intubated) infants [11, 31].

Studies varied by characteristics of the intervention, the outcome of interest, and how the outcome of interest was measured. Therefore, below we will briefly discuss characteristics of intervention, parent-centered outcomes, and outcome assessments, and we will synthesize results based on the effect of the intervention as measured by particular outcome assessments as well as synthesize results based on the outcomes of interest.

### Intervention characteristics

Intervention characteristics also differed across studies. Eight of the studies examined IM as a component of a larger developmental intervention, and 5 studies examined IM as an exclusive intervention [22, 23, 26]. Length of intervention ranged from 24–48 h [22, 23] to several weeks [11, 24, 25, 29, 31, 32]. See Table 1 for additional study characteristics.

### Parent-centered outcomes

Six primary parental outcomes related to IM emerged: 1) anxiety (*n* = 7), 2) perceived stress (*n* = 4), 3) depressive symptoms (*n* = 7), 4) maternal-infant interaction (*n* = 5), 5) maternal satisfaction *n* = 3), and 6) maternal competence (*n* = 2).

### Outcome assessment characteristics

Despite shared outcomes of interest, many different outcome measures were used across studies. For parent anxiety, the most commonly used assessment was the “state” section from the State Trait Anxiety Index (STAI). Of the 5 studies that used this outcome measure, 2 of the interventions were comprised of massage only [22, 23] and the other 3 used massage as part of a larger multisensory intervention [11, 24, 29]. Other anxiety outcome measures used included the Profile of Mood States (POMS) anxiety subscales [28], the PROMIS Anxiety Short Form [28], and the Worry Index [24].

In regards to stress, different outcome measures were used depending on the study, on the timing of the assessment, and the quality of stress. For perceived stress during the NICU period, he Parental Stress Scale: NICU (PSS:NICU), which is tailored to the NICU environment and infant acuity was used in 4 studies [11, 19, 27, 29], and the Parental Stress Scale: Prematurely Born Child, another version of this scale, was used in another study [24]. The Perinatal Post Traumatic Stress Disorder Questionnaire (PPQ) was also used in 2 studies [11, 29], and the Life Stress Subscale of the Parenting Stress Index (PSI) was used in one study [11].

Regarding depression as an outcome, 3 studies that examined massage intervention exclusively, used the Profile of Mood States (POMS) (*n* = 2) ([23, 28] or the Beck Depression Inventory (BDI) (*n* = 1) [26]. Outcome measures for the 4 studies examining massage as a component of a longer multisensory intervention varied widely and included the Centers for Epidemiological Studies Depression Scale (CESD) (*n* = 1), the Edinburg Postnatal Depression Scale (EPDS) (*n* = 2), the BDI (*n* = 2), and the PROMIS Depression Short Form (*n* = 1).

### Risk of bias in included studies

Eight of the 13 included studies were rated “high risk” in 4 or more categories using the RoB:2 revised Cochrane risk-of-bias tool. Due to the very nature of the infant massage intervention, blinding of participants to the intervention was only achieved in one study [11]. The majority of studies were scored “unsure risk” in at least one category because a quality assessment category was not specifically addressed in the manuscript. Small to moderate samples sizes in all but 4 studies [24, 25, 31] as well as quasi-experimental designs [29] or lack of comparison group [27] in other studies limited the generalizability of results.

### Synthesis of results by effect size

Because of high variability in the type, duration, and approaches to infant massage intervention, variability of outcome measures used, limited numbers of studies that met inclusion criteria, and search strategies, we chose to perform a scoping review. For studies where both group means and standard deviations were available, we calculated effect size of the intervention (Table 3). Studies were categorized as having small (d ≤ 0.2), moderate (d ≤ 0.5), or large (d ≥ 0.80) effect sizes based on Cohen’s d [34]. Based on effect size calculations, all 4 studies that examined the impact of IM either as a stand-alone or combined multisensory intervention on maternal anxiety had small (< 0.2) effect sizes [22, 23] or no effect [11, 29]. The interventions in studies by Matricardi et al. [19] and Pineda et al. [11] showed moderate to large effect sizes based on the PSS:NICU outcome measure of perceived stress. The effect size of the intervention in the Lotfalipour et al. study [28] was large for the POMS, a measure of maternal depression.

**Table 3:** Overall evidence of the effect of infant massage intervention (short-term)

First author, year	Anxiety		Perceived Stress		Depressive Symptoms	
	Outcome Measure	Effect Size^a^	Outcome Measure	Effect Size^a^	Outcome Measure	Effect Size^a^
**Afand et al. 2017** [22]	STAI	0.125				
**Feijo et al. 2006** [23]	STAI	0.113			POMS	-0.026
**Lotfalipour et al. 2019** [28]					POMS	0.928
**Matricardi et al. 2013**^**b**^ [19]			PSS:NICU, SS PSS:NICU, IBA PSS:NICU, PRA	0.63667 0.2975 1.1537		
**Pineda et al. 2021 **[11]	STAI, State subscale	0.015	PSI PSS:NICU	0.007 0.48480931	EPDS	0.019
**Pineda et al. 2020 **[29]	STAI, State subscale	0.014	PSS:NICU, PRA PSS	0 0.0122	EPDS	0.059

*STAI* State-Trait Anxiety Inventory, *POMS* Profile of Mood States, *PSS:NICU* Parental Stress Scale:Neonatal Intensive Care Unit, *SS* Sights and Sounds subscale, *IBA* Infant Behavior and Appearance subscale, *PRA* Parental Role Alteration subscale, *PSS* Parental Stress Scale, *EPDS* Edinburgh Postnatal Depression Scale

^a^small (d ≤ 0.2), moderate (d ≤ 0.5), or large (d ≥ 0.80) effect sizes based on Cohen’s d

^b^Reporting scores for mothers only, not fathers included in this study

### Synthesis of results by parent outcomes

#### Anxiety

A total of 7 studies examined the impact of maternally-administered IM on measures of parental anxiety. Three of these studies examined maternal anxiety of interventions comprised exclusively of massage techniques. Feijo et al. [23] randomized 40 mothers into 2 groups: one that learned and performed IM, and one that observed their infant being massaged. The researchers found that only the group performing massage demonstrated a significant reduction in scores in the “state” portion of the State-Trait Anxiety Inventory (STAI) (intervention group pre = 39.2(7.3), post = 27.9(7.1), *p* < 0.05; observation group pre = 34.9(7.4), post = 33.7(7.2), *p* > 0.05. Afand et al. [22] and Lotfalipour et al. [28] examined short-term IM (over 24–48 h and 5 days respectively) using experimental designs. Afand et al. used the “state” portion of the STAI to characterize maternal state anxiety over a 24–48 h period immediately postpartum. They found that the massage intervention group demonstrated significantly lower STAI scores (27.46 (6.17)) than the control group (32.46 (6.54)) at hospital discharge; however, both groups demonstrated significant improvements in state anxiety from the initial postpartum interview to discharge, 24–48 h later (*p* < 0.001) [22]. Loftalipour used the Profile of Mood States (POMS) anxiety subscales and found a significant reduction in symptoms for the intervention group after a 5 day massage intervention. These scores were not reported. Furthermore, intervention and control groups in this study were significantly different by maternal education and age, potentially biasing results [28].

Four studies examined intervention bundles that included IM and associated changes in maternal anxiety. Holditch-Davis et al. [24] randomized 240 mothers into 3 groups, 1) kangaroo care (ie., skin-to-skin holding), 2) ATVV, or 3) control, and found that mothers in the ATVV did not demonstrate significant differences in anxiety based on STAI scores over the course of hospitalization. This study also used the Worry Index, a survey designed to measure how much mothers worry about their infant’s risk for health issues and found that these scores declined over time for all groups, with no significant difference between groups (ATVV, kangaroo, and control). Group means and standard deviations were not reported for each group, nor p values for outcomes that did not reach *p* < 0.05. Pineda et al. [29] compared historical controls to a prospective group of mother-preterm infant dyads who participated in the “Supporting and Enhancing NICU Sensory Experiences” SENSE intervention, but did not find any differences between groups in measures of maternal anxiety using the “state” section of the STAI (control = 30.1 (8.5), intervention = 28.0 (8.6) *p* = 0.36 [29]. In 2021, Pineda et al. [11] published an RCT of the SENSE Program and used the STAI to measure anxiety. Group differences at term equivalent age were as follows: (STAI control 38.5 (11.9), STAI intervention = 35.1 (17.9) *p* = 0.62). Letzkus et al. [27] evaluated the feasibility of a maternally-administered developmental bundle, which included massage, for infants born less than 1500 g. Using the PROMIS anxiety scale in a small cohort of 11 mothers, no significant differences were appreciated between pre- and post-intervention scores (pre intervention = 15.3 (1.4), post intervention = 12.4 (1.4), *p* = 0.16) [27].

#### Perceived stress

Four studies examined parental perceived stress in relation to parent-administered IM. In the RCT by Holditch-Davis et al. [24], mothers in the ATVV group demonstrated significant improvements in measures of stress based on the Parental Stress Scale: Prematurely Born Child (PSS:PBC) (*p* < 0.001). Group means and standard deviations were not reported for each group, nor *p* values for outcomes that did not reach *p* < 0.05. Matricardi et al. [19] conducted a RCT of 42 parent couples, mothers and fathers, of infants born < 32 weeks gestation. The intervention group received education about their infant’s behavior and massage education, and the control group received standard care. While participation in massage intervention reduced stress from birth to hospital discharge in both mothers and fathers based on the Parental Stress Scale:NICU (PSS:NICU) in the subscale of “infant appearance and behavior” (t (41) = 2.56, *p* = 0.014)), but scores increased in the standard support group, (t (41) = 2.71, *p* = 0.010). Additionally, the intervention group parents reported lower role-stress between birth and hospital discharge (t (41) = 4.31, *p* = 0.000) [19].

In the pilot study by Pineda et al. [29] examining the SENSE program, no significant differences between groups in measures perinatal post-traumatic stress at term equivalent age based on the PPQ (control = 8.25 (7.6), intervention = 6.23 (7.6), *p* = 0.33) were appreciated. In the 2021 RCT examining SENSE, Pineda et al. [11] used the PSS:NICU to examine perceived stress at term equivalent age and found no group differences after controlling for medical factors (ionotropic support, patent ductus arteriosus, necrotizing enterocolitis, parenteral nutrition > 21 days, mechanical ventilation > 7 days, bronchopulmonary dysplasia, grade III-IVH intraventricular hemorrhage, or periventricular leukomalacia) and social factors (based on a social risk score not defined) (control = 3.1 (1.2), intervention = 2.5 (1.0) *p* = 0.28). This study also used the Life Stress Subscale of the Parenting Stress Index (PSI) to measure perceived stress, and the MPQ to measure post-traumatic stress both at term equivalent age and at one year follow up, but no significant between group differences were appreciated. Group differences at term equivalent age were as follows: (PSI control = 60.5 (13.7), PSI intervention = 58.4 (20.7) *p* = 0.56; MPQ control = 12.0, MPQ intervention = 8.0, *p* = 0.96). Group differences at one-year follow up were as follows: (PSI control = 57.0 ± 19.6 PSI intervention = 52.7 ± 24.0, *p* = 0.44, MPQ control = 13.0, MPQ intervention = 10.5, *p* = 0.79). In the small cohort pilot study conducted by Letzkus et al., 11 mothers who participated in a developmental bundle which included massage demonstrated significantly improved stress levels based on the PSS:NICU from baseline to hospital discharge pre intervention (7.4 (0.8)) to post intervention (5.7 (0.7), *p* = 0.02)).

#### Depressive symptoms

Seven studies examined measures of maternal depression related to maternally-administered IM. Three of these studies examined massage exclusively. In the study by Feijo et al. [23] described above, both groups of mothers either randomized to administer or observe massage demonstrated significant reductions in depressive symptoms immediately post-massage based on the POMS (pre intervention group = 2.4 (3.0), post intervention group = 1.0 (2.1), *p* < 0.05; pre observation group = 2.5 (2.9), post observation group = 0.9 (1.8), *p* < 0.05. A study by Lotfalipour et al. [28] comparing massage and control groups demonstrated a significant improvement in POMS scores after 5 days of intervention for the massage group only (intervention = 118.92 (3.45), control = 141.73 (6.1), *p* = 0.005. In a small RCT of 12 dyads, maternal depressive symptoms based on the Beck Depression Inventory (BDI) were reduced from baseline (control = 10.2, (9.6), massage = 13.4 (7.3)) to 7 days (control = 6.0 (4.3) massage = 9.2 (4.8) for both intervention and control groups [26]. Group differences were not analyzed for this study, however, due to small cohort sizes.

Four studies that examined IM as a component of a developmental intervention throughout infant hospitalization demonstrated inconsistent results related to maternal depressive symptoms. When comparing kangaroo care, ATVV, or control groups, Holditch-Davis et al. [24] found that mothers in the ATVV group demonstrated more rapid decline and leveling off of depressive symptoms based on the Centers for Epidemiological Studies Depression Scale (CESD) than other groups; yet, Pineda et al. found no differences between intervention and control groups for mother’s depressive symptoms at term equivalent age based on the Edinburgh Postnatal Depression Scale (EPDS) in a 2020 cohort comparison study (historical controls = 7.08 (4.2), intervention = 8.5 (5.9), *p* = 0.27) [29] and a 2021 RCT (control = 9.0 (4.7), intervention = 8.5 (5.5), *p* = 0.08) [11] The 2021 RCT also examined maternal depressive symptoms at 1 year corrected age using the BDI and found no group differences (control = 3.6 (4.1), intervention = 3.9 (5.9), *p* = 0.96). The above studies, however, differed in methods for tracking the fidelity and frequency of maternally-administered interventions, with mothers exclusively providing developmental intervention in the study by Holditch-Davis [24] and with parents *and* researchers providing the developmental intervention in the two studies by Pineda et al. [11, 29] In the Letzkus et al. study [27], a small single cohort of mothers (*n* = 11) who participated in a maternally-administered developmental bundle had significantly reduced scores on the PROMIS depression scale from pre-intervention (11.1 ± 0.9) to post-intervention (9.0 ± 0.5, *p* = 0.002).

#### Mother-infant interaction

Five studies examined measures of maternal-infant interaction. Mother-infant interaction was examined in various contexts: during IM (*n* = 1) [32] over a 5-day intervention period (*n* = 1) [30] over a period of several weeks [25] (*n* = 1), while the mother fed the infant (*n* = 1) [31], and during mother-infant play (*n* = 1) [31]. White-Traut et al. [32] completed an analysis of 36 videos of kangaroo care or ATVV sessions that took place during the Holditch-Davis et al. study [24]. Data analysis revealed that significantly more engagement and disengagement behaviors were noted in the ATVV group than the kangaroo care group; therefore, authors determined that ATVV creates greater opportunity for infant and mother to establish a pattern of reciprocal interaction [32]. In 2013 White-Traut et al. [31] published results of an RCT that examined the impact of H-HOPE on maternal-infant interaction, using the Nursing Child Assessment Satellite Training-Feeding Scale (NCAST) during breast or bottle feeding and the Dyadic Mutuality Code (DMC) during mother-infant play. For both feeding and play, the H-HOPE group demonstrated marginally better positive interactions than controls, but the differences did not reach significance [31]. Shoghi et al. [30] completed a small RCT comparing measures of maternal-infant attachment throughout a 5-day IM intervention between massage (*n* = 20) and control (*n* = 20) groups and found a significant post-intervention effect in the intervention group. Using the HOME inventory (Home Observation for Measurement of the Environment), an outcome measure related to maternal-infant interaction, Holditch-Davis et al. [24] found that mothers who regularly massaged their infants during these periods provided a more positive home environment at 2 and 6 month follow-up than controls.

#### Maternal satisfaction

Maternal satisfaction was measured in 3 studies through surveys developed by the researchers pertaining to individual projects. Holditch-Davis et al. administered a satisfaction survey and found that mothers in the ATVV group demonstrated significantly higher changes post-intervention in response to the prompt: “learn new ways to stimulate and teach my infant,” but no differences were found between groups for the following prompts: whether the mother would recommend the study to others, the degree to which [they] changed as a person, and the degree to which [they] changed as a mother [24]. Feijo et al. [23] also administered a parent satisfaction survey that revealed that both groups of mothers – those who administered massage and those who observed – believed their infant enjoyed massage and therefore, did not demonstrate significant differences between groups. Livingston et al. [26] also described positive maternal satisfaction with the massage program, but group comparisons were not made due to small sample size.

#### Maternal competence

Only 2 studies examined parent perceived competence. Pineda et al.’s pilot study of SENSE [29] found that mothers in the intervention group experienced significantly improved maternal confidence as compared to historical controls, but these results must be interpreted with caution considering the time gap between cohorts that could introduce several confounding factors. Pineda et al.’s 2021 RCT [11] demonstrated higher maternal confidence scores in the SENSE group, but the relationship failed to reach significance after controlling for medical and social factors (described above) [11].

## Discussion

Taken together the result of this scoping review suggest that maternally-administered IM may have positive short-term effects on maternal anxiety and stress [22, 23, 28], but there is limited evidence to support its effectiveness in reducing maternal anxiety and stress throughout hospitalization and follow-up periods [11, 19, 24, 29]. Based on effect size calculations in small study cohorts, maternally-administered IM may have a moderate to large effect size on maternal perceived stress [11, 19] and depressive symptoms [28]. Maternal depressive symptoms were reduced over a short period of time through maternal administration or observation of massage [22, 23, 28] and mothers who massaged their infants throughout hospitalization demonstrated more rapid declines and leveling off of depressive symptoms than other groups [24]. Measures of maternal-infant interaction between preterm infants and their mothers seem to improve over short-term periods using IM [30–32]and is associated with improved home environment at 2 and 6 month follow-up [24]. Mothers who learned massage were more likely to report that they had “learned new ways to stimulate their infant” [25], and overall, reported being satisfied with massage intervention [23, 26]. Multisensory interventions that include massage may also improve maternal sense of competence [11, 29].

Maternal mental health, especially in the NICU, can be influenced by many factors, such as infant health acuity, maternal baseline mental health, social support structures, and other situational or complex social issues [5]. Therefore, while anxiety, stress, or depressive symptoms may be alleviated transiently with IM, it may be more difficult to parse out the impact of IM on maternal mental health over long periods of time. In addition to these potential confounding factors, the frequency of maternally-administered IM may be influenced by work or home demands or the infant’s response to massage, which changes the dosage and potential for influence. As the infant grows and develops in the NICU, parent goals shift from holding and interacting with the infant to practicing bottle and/or breastfeeding in order to prepare for discharge home. This shift in focus is appropriate and aligns with infant maturity and social behaviors [35]. Infant maturity demonstrating readiness to feed often coincides with the infant’s ability to meaningfully engage in IM, and may compete for the mother’s time spent at the bedside.

Increased quality of maternal-infant interaction was observed post-IM at various time points and with various activities – during holding after 5 days [30], at 6 weeks corrected age during feeding [31], at 6 weeks corrected age during play [25], during weekly massage sessions during hospitalization [32],  and at 2–6 months post-discharge [24]. Once the infant demonstrates neurobehavioral maturity and readiness for massage, IM presents more opportunities for engagement as compared to skin-to-skin holding because of the reciprocal interaction taking place between the mother’s actions and the infant’s response during massage [28]. Along with deep pressure tactile input provided through massage strokes, maternally-administered IM incorporates visual and auditory stimulation as the mother changes facial expressions and talks to the infant, establishing early reciprocity [32]. The infant’s positive responses of increased body relaxation [7, 32] or increased visual engagement [32] gives the parent “in the moment” feedback about their performance. Therefore, learning IM with sensitivity to infant cues provides the parent with a meaningful activity in which they can actively the observe benefits of spending time with their infant.

Other outcome measures included in this review attempted to quantify the mother’s satisfaction and sense of parenting competence. It is well-described that mothers generally feel helpless in their ability to care for their preterm infant, especially in the earliest stages of the NICU stay [4]. Learning safe and effective hands-on interventions can empower the mother and build confidence not just in IM administration, but potentially in other parenting skills. Based on studies reviewed, therapists, nurses, and developmental specialists can teach parents infant massage on medically stable infants as early as 32–34 week postmenstrual age [11] to support parent engagement in bedside care and to provide a foundation for developing more complex parenting skills over time.

### Limitations

It was difficult to draw conclusions about IM effectiveness in this scoping review due to the variability in intervention approach, administration, and frequency between studies. When possible, effect sizes were calculated to better understand the impact of the intervention between groups; however, no effect sizes were reported in these studies, and means and standard deviations were only reported for 6 of 13 studies included in this review. Additionally, outcome measures for anxiety, stress, depression, satisfaction, interaction, and competence varied greatly across studies, making collective assessment of the impact of IM on outcomes challenging.

Studies included in this review examined IM as a stand-alone intervention and as part of a larger multisensory intervention, limiting generalizability of findings. Furthermore, study methodological rigor was lacking in most of the studies included in this review. One large 3-arm RCT of 240 mother-infant dyads representing 2 distinct geographical regions of the U.S. collected a large number of outcome measures that were reported in 3 separately published manuscripts [24, 25, 32]. Authors note this as a potential design limitation that could lead to Type I error in reporting and limit generalizability. However, to date, this study is the largest RCT to primarily examine maternal outcomes related to IM. Six studies, because of small sample size or design, must be interpreted with caution due to high risk for bias [11, 23, 26, 27, 29, 31].

### Areas for future research

Additional research examining maternally-administered IM is warranted to extend and validate the findings described in this scoping review. Definitive conclusions about the impact of preterm infant massage on the parents that administer it are limited based on small sample size, poor quality, and insufficient effect size reporting. It is clear that IM has benefits for the preterm infant population [7]; however, it should be determined if parent-administration of massage at recommended frequencies is feasible and equally effective – both for infant-centered outcomes and parent-centered outcomes. This research focus will inform future institutional staffing and policy shifts necessary to support IM uptake in NICUs.

It is important to address in future research the mechanism by which massage may improve parent outcomes. Because massage has been examined both as a stand-alone intervention and part of a larger multisensory intervention, it is not possible to clearly discern the role that IM may play in the parent’s response. For example, does the act of administering IM influence the parent on a biological level? Emerging evidence suggests that maternal-infant dyadic interaction may reduce salivary cortisol, a biomarker for stress, in infants [36, 37]. Vittner et al. found that skin-to-skin holding over a period of one hour in the NICU results in increased oxytocin levels in mothers, fathers, and infants and decreased cortisol in the infants [38]. These changing hormonal levels were also associated with improved responsiveness and synchrony in the parent-infant relationship as measured by videos of parent-infant interaction [38]. While White-Traut et al. [37] observed reduced cortisol in healthy *fullterm* infants following Massage + intervention, these biomarker outcomes have not been measured in preterm infants or their parents.

Programs that incorporate IM may also influence parent outcomes because of the educational component that supports dyadic interaction. For example, most multisensory programs included in this review include education about infant cues and parent responsiveness – both of which can be enhanced and reinforced through maternally-administered IM. A better understanding of the potentially different mechanisms of action would lead to improved precision of IM intervention and application in the clinical setting.

Another potential area for expansion in this intervention is to include family members beyond the birth parent. While prevalence of stress and depression in fathers of NICU infants is well-studied, the majority of intervention studies examined for this scoping review focused on improving maternal outcomes. Furthermore, very limited data is available about the mental health of same-sex parent partners in the NICU [39]. Only one study in this review included fathers in the developmental intervention [19]. While this study found that infant massage appears to have different effects on mothers and fathers, future studies should examine the role and benefits of infant massage administered by fathers and same-sex partners.

## Conclusions

In conclusion, this scoping review explores evidence linking maternally-administered infant massage to reductions in anxiety, stress, and depressive symptoms in mothers of hospitalized preterm infants; and improvements in maternal-infant interactions, maternal satisfaction, and maternal competence in the short-term. Over time, IM appears to be associated with increased maternal confidence and a more positive home environment and may reduce stress and depressive symptoms in the post-natal period, Additional research with larger cohorts, employing more rigorous methodology, and incorporating more widespread outcome measures is needed to study IM and its associations with parent outcomes. Researchers should develop targeted and standardized IM interventions that facilitate parent-infant interaction, reduce known barriers to parental presence in the NICU, and examine feasibility of implementing parent education in IM as standard of care practice in NICUs. Study populations should be expanded to include fathers, partners of the birth parent, and other members of the family unit.

## Data Availability

All data generated or analyzed during this study are included in this published article.
